# Wellness Activities, Stress, and Academic Performance in Medical Students

**DOI:** 10.7759/cureus.62704

**Published:** 2024-06-19

**Authors:** Kian Habashi, Edward Simanton

**Affiliations:** 1 Medical Education, Kirk Kerkorian School of Medicine at the University of Nevada, Las Vegas, Las Vegas, USA

**Keywords:** clerkship, pre-clerkship, wellness activity, academic performance, student stress, student wellness

## Abstract

Introduction: Medical student wellness has a range of effects from academic performance to tragic mortality. Many factors correlate with academic performance, including study environments, faculty support, research participation, and student attitude. Its relationship with student stress and wellness demonstrates mixed results. This study hopes to help clarify these results and will also assess the interplay of these factors in relation to the pre-clerkship and clerkship phases of medical school.

Methods: This retrospective descriptive study was conducted using a de-identified database from 2017 to 2023, provided per an Institutional Review Board (IRB) protocol. The subjects were the students of the classes of 2021-2027 at the Kirk Kerkorian School of Medicine at UNLV. The database included performance data including semester, clinical subject, and USMLE Step 1 and Step 2 exam scores. Other data included variables relating to self-perceived stress and time spent in wellness activities before medical school and at the end of the pre-clerkship and clerkship phases of medical school. The effects of these variables were calculated with bivariate correlations and independent samples T-tests using cut-off points calculated from the class means of those variables. A total of 361 medical students were included in the study.

Results: Students with high wellness time had lower stress levels in both the pre-clerkship and clerkship phases of medical school (5.83 vs. 7.3 p < 0.001^*^ and 5.74 vs. 8.49 p < 0.001^*^, respectively). Students with low stress levels in the pre-clerkship phase scored 5.81 points higher on the Step 1 exam (p = 0.013^*^). Clerkship phase stress levels were significantly negatively correlated with all clinical subject exams except for pediatrics. Stress levels in the pre-clerkship and clerkship phases had similar relationships with the second and third semester and Step 2 exams, respectively, although not statistically significant. Wellness activity time did not have a significant relationship with academic performance.

Conclusion: Stress levels had significant negative relationships with many medical school exams. Although wellness activity time did not have a direct relationship with academic performance, its relationship with stress levels can allude to an indirect effect on academic performance. This, along with fending off burnout and stress, are reasons why medical student wellness should be a priority for medical schools, faculty, and their students.

## Introduction

Stress is all too common in America, with 24% of the population reporting high stress levels in 2023 per the American Psychological Association [1]. This is especially true for medical students, who have extreme academic and time demands [2]. Studies have estimated that almost half of all medical students experience some level of burnout and depression, and this finding increases with each subsequent year in medical school [3,4]. Medical student stress and burnout have been a well-known issue for decades and are still very present today [3,5]. This issue has a range of effects from academic performance to tragic suicide [5]. As such, it is an important concern for students, faculty, and administration.

Many studies have established the lack of wellness in medical students, the barriers to wellness, and avenues to improve it [3,5]. There are many contributing factors to medical student burnout and stress, including academic rigor, lack of sleep, and the witnessing of patient sickness and mortality [5-8]. A medical student’s own tendency toward perfectionism may also predict burnout [9]. Stress is also more prevalent in students experiencing loneliness and a lack of social support and networks [10]. Some medical schools have already implemented programs to help resolve this issue and enhance the wellness of their students. Dedicated sessions and handouts encouraging healthy activities and relationships and on how to decrease stress and burnout were implemented at Vanderbilt School of Medicine [11].

Many factors correlate with student academic performance, including study environments, faculty support, and involvement in independent research [12]. Some other obvious contributing factors are study time, motivation, and attitude [13]. Time allocated to quiet, independent study and less time on social networking sites also contribute to academic success [14]. Studies on the relationship between medical student stress and academic performance have shown mixed results. In an older study, Stewart et al. demonstrated a significantly negative correlation, whereas Ruiz et al. found no relationship [5,10]. Ruiz et al., however, found that medical student burnout, a factor related to stress, had a negative relationship with exam scores [10]. One study by Toews et al. even found that some students believe their stress actually enhances academic performance [15].

There is still a lack of current studies examining the relationship between wellness activities, stress, and academic performance in medical students. Additionally, no studies have assessed this relationship regarding the pre-clerkship versus clerkship phases of medical school. In this study, we seek to determine the relationship between those factors in medical students at the Kirk Kerkorian School of Medicine at the University of Nevada, Las Vegas (UNLV) by analyzing anonymous self-reported survey scores and exam data. This study can bring further awareness of the interplay of these factors and therefore extend the scope and knowledge of the importance of student wellness. It can offer more reasons to provide wellness resources and education.

## Materials and methods

This retrospective descriptive study was conducted using a de-identified database of program evaluation data, provided in accordance with approved Institution Review Board (IRB) protocol number UNLV-2022-293 “Medical Education Research Using Program Evaluation Data.” The data included in the study was collected from 2017 to 2023 and represents the classes of 2021-2027. The database included performance data including the semester exam, clinical subject exam, and the United States Medical Licensing Examination (USMLE) Step 1 and Step 2 exam scores. Other data included variables relating to self-perceived stress and time spent in wellness activities prior to medical school and at the end of the pre-clerkship and clerkship phases of medical school. The subjects included in the database were students of the Kirk Kerkorian School of Medicine at the University of Nevada, Las Vegas (UNLV). Inclusion criteria for subjects included that they must be a current medical student at Kirk Kerkorian School of Medicine at UNLV at the time of data collection. Exclusion criteria were any subject that was not a current medical student at Kirk Kerkorian School of Medicine at UNLV at the time of data collection.

The self-perceived stress variable came from the Perceived Stress Scale 4, a classic stress assessment instrument developed by Cohen et al. in 1983, trialed for reliability (Cronbach alpha = 0.80), and utilized by the Association of American Medical Colleges [16]. A larger number on the scale defines a higher perceived stress. The wellness variable came from the question: “In an average week, how many hours do you spend on your own wellness?" Wellness activities were defined by the student and could be anything from weightlifting, to getting a massage, or painting. The effects of these variables were calculated with independent samples t-tests using cut-off points calculated from the class means of the number of hours spent in wellness activities per week and perceived stress scale scores for the pre-clerkship and clerkship phases of medical school. Below the cut-off point was reported as “low-stress levels” or “low wellness time,” and above the cut-off point was reported as “high-stress levels” or “high wellness time,” These values are reported in Table 1.

**Table 1:** Mean number of hours spent in wellness activities per week and perceived stress scale scores Stress scale score range: 0-16 *N, the number of respondents, varies due to the exclusion of subjects that did not answer the particular survey item.

	Mean	“Low”	“High”	Standard deviation	N
Pre-clerkship phase wellness time	8.45	<8.4	>8.4	4.77	173*
Pre-clerkship phase stress scale score	6.75	<6.7	>6.7	3.03	296*
Clerkship phase wellness time	6.65	<6.6	>6.6	4.35	177*
Clerkship phase stress scale score	7.04	<7	>7	3.25	231*

Responses with missing data and incomplete responses were removed from collection prior to data analysis. These responses may have been subject to self-reporting and social-desirability reporting bias. The anonymity of the responses was guaranteed in hopes of reducing this. Sampling bias may have also occurred but was minimized by distributing the survey link to all students’ school-verified emails. Steps were taken to standardize the questionnaire used for the study: target respondents and methods utilized to reach respondents were selected, questionnaire items were appropriately worded, and face and content validity were verified prior to administration.

Statistical analysis

Bivariate correlation tests and independent samples t-tests were utilized for data analysis. Two-tailed significance values were calculated for each test. IBM SPSS Statistics for Windows, Version 29 (Released 2021; IBM Corp., Armonk, New York, United States) was used for data analysis.

## Results

A total of 361 medical students from Kirk Kerkorian School of Medicine were surveyed. The demographic characteristics of the study sample are described in Table 2.

**Table 2:** Descriptive characteristics of the study sample Total number of respondents: 361 Mean length of years between graduation and matriculation into medical school: 1.90 (SD = 2.26) ^*^First-generation student was defined as a subject who is the first of their family to pursue higher education above a high school diploma.

Descriptors	Percentage of sample	N
Age <23	26.6	96
Age 23-25	40.2	145
Age 26-29	23.3	84
Age 30+	9.4	34
Missing age	0.6	2
Female	51	184
Male	49	177
White	47.6	172
Hispanic	16.1	58
Asian	29.1	105
Black	5.0	18
Other	1.9	7
First-generation student^*^	28.5	103

We first desired to analyze if the number of hours students spent in wellness activities per week at baseline correlated with the number of hours that they spent in wellness activities in each of the subsequent phases of medical school. Pearson correlation coefficients were calculated for each analysis and two-tailed significance values were provided. Significance values at the 0.05 level are noted with ^*^. Table 3 shows those results.

**Table 3:** Hours spent in wellness activities at baseline correlated with hours spent in wellness activities in subsequent phases of medical school Significance values at the 0.05 level are noted with ^*^. N, the number of respondents, varies due to the exclusion of subjects that did not answer both of the survey items in the particular correlation analysis.

	Pre-clerkship phase	Clerkship phase
Baseline	r = 0.334; p < 0.001^*^; N = 112	r = 0.360; p = 0.009^*^; N = 52

As expected, the amount of hours students spent in wellness activities per week at baseline significantly correlated with the hours that they spent in wellness activities in subsequent phases of medical school.

**Figure 1:**
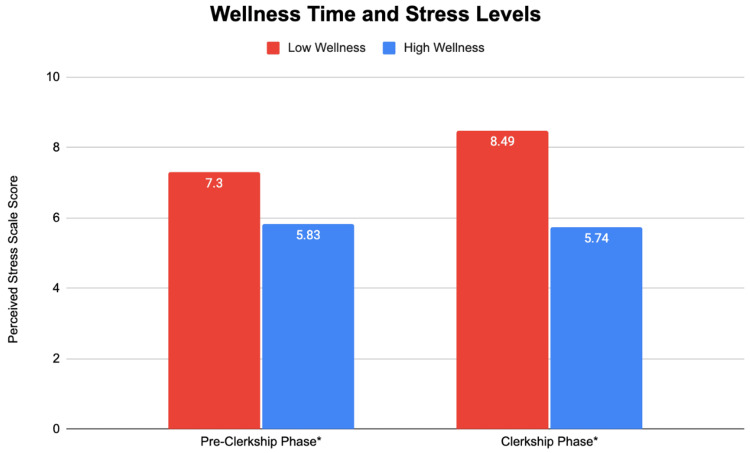
Wellness time and stress levels in the pre-clerkship and clerkship phases of medical school Significance values at the 0.05 level are noted with ^*^. Pre-clerkship phase: t = -3.545; df = 171; p < 0.001^*^ Clerkship phase: t = -6.056; df = 174; p < 0.001^*^

We sought to find if there was a difference in stress levels of medical students when they had a high or low wellness time in each phase. Figure 1 illustrates those results.

Unsurprisingly, our results show that medical student stress levels are significantly lower when they spend more time on wellness activities during both the pre-clerkship and clerkship phases of medical school. The vice versa is also true. This relationship may be stronger in the clerkship phase of medical school.

**Figure 2:**
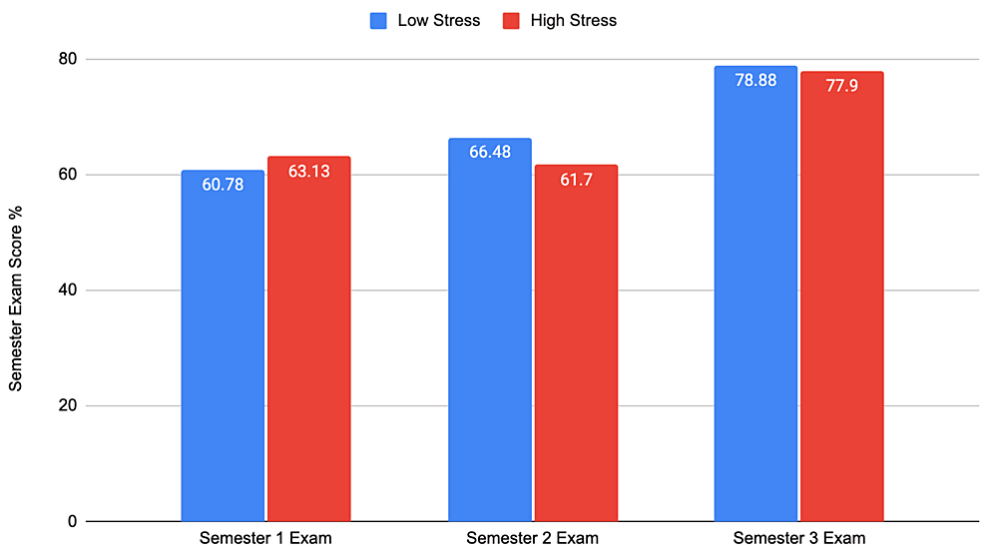
Pre-clerkship stress levels and semester exam performance Semester 1: t = 0.564; df = 177; p = 0.573 Semester 2: t = -1.144; df = 177; p = 0.254 Semester 3: t = -0.308; df = 177; p = 0.758

**Figure 3:**
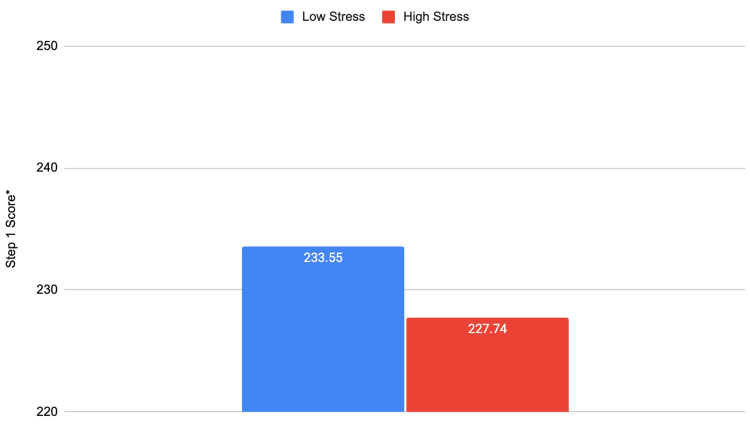
Pre-clerkship stress levels and Step 1 exam performance Significance values at the 0.05 level are noted with ^*^. Step 1: t = -2.510; df = 182; p = 0.013^*^

We then assessed how high and low stress levels during the pre-clerkship phase were related to the semester exam and Step 1 exam scores, the exams taken during and at the end of that phase, respectively. The results are shown in Figures 2, 3.

We found that pre-clerkship phase stress levels do not have a significant effect on semester exam performance. However, stress levels did have a significant relationship with Step 1 exam performance. Medical students with lower stress had on average a 5.81 higher Step 1 score than those with higher stress.

**Figure 4:**
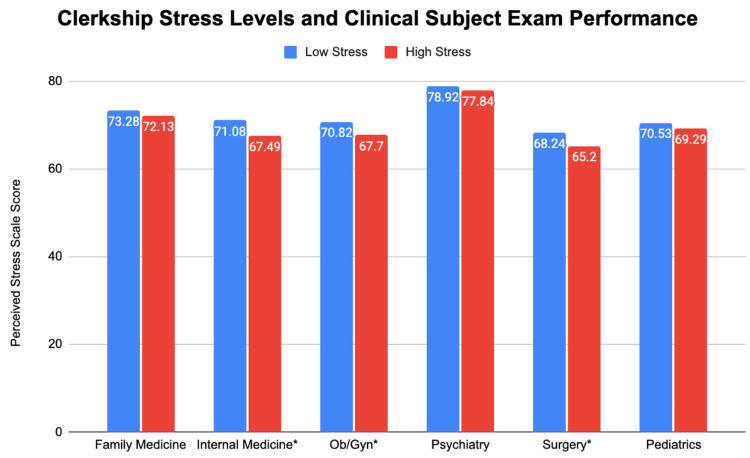
Clerkship stress levels and clinical subject exam performance Significance values at the 0.05 level are noted with ^*^. Family medicine: t = -1.138; df = 226; p = 0.256 Internal medicine: t = -2.717; df = 226; p = 0.007^*^ Obstetrics and gynecology (Ob/Gyn): t = -2.515; df = 224; p = 0.013^*^ Psychiatry: t = -1.173; df = 226; p = 0.242 Surgery: t = -0.2588; df = 224; p = 0.010^*^ Pediatrics: t = -0.914; df = 226; p = 0.361

**Figure 5:**
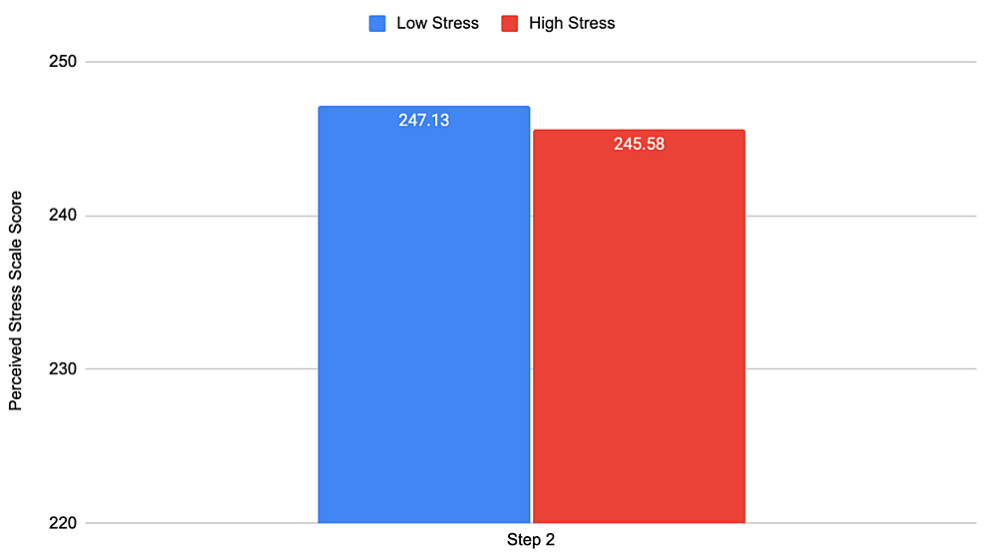
Clerkship stress levels and Step 2 exam performance Step 2: t = -0.690; df = 168; p = 0.491

Then, we assessed the relationship between stress levels to academic performance in the clerkship phase of medical school. We analyzed its relationship with clinical subject exams and Step 2, the tests taken in the middle and end of Phase 2, respectively. Figures 4, 5 demonstrate these results.

We found significant relationships between lower stress levels in the clerkship phase of medical school and higher clinical subject exam scores in internal medicine, ob/gyn, and surgery. All other clinical subject exams and Step 2 scores exhibited this same trend but did not achieve significance. However, we did find that the family medicine and psychiatry clinical subject exams had significant negative correlations with stress levels during the clerkship phase (r = -0.133^*^; p = 0.045; r = -0.139^*^; p = 0.036, respectively).

## Discussion

Our study suggests that if students have a higher time commitment to wellness activities before medical school, they are more likely to sustain those habits throughout medical school. Medical students often have trouble concerning time management, which may make it difficult to prioritize these wellness activities while they are already in medical school [17]. This is especially true considering that many of the ways medical students combat stress like physical exercise, playing sports, and socializing usually require a substantial amount of time [15,17].

The results of this study demonstrate that the number of hours medical students at the Kirk Kerkorian School of Medicine spend a week on various wellness activities has a significant negative relationship with the amount of self-reported stress that they perceive. This is similar to the findings from Hill et al. and Ruiz et al. [10,17]. Hill et al. found that students with lower reported stress more frequently exercise, read, and participate in sports and extracurricular activities [17]. However, other wellness activities such as sleeping, listening to music, playing with pets, and cooking did not relate to the stress levels in their medical student cohort [17]. Additionally, certain wellness activities modulate stress depending on the nature of that activity, the higher the intensity of exercise performed by medical students, the lower their stress, according to a study by Leuchter et al. [18]. The nuance in wellness activity type, frequency, and intensity is an important consideration for medical students attempting to lower their stress levels.

Additionally, we found that stress levels in the pre-clerkship and clerkship phases of medical school have negative relationships with Step 1 and most clinical subject exam scores, respectively. These findings echo similar results in older studies that found negative correlations between stress and academic performance [5]. This is also in line with findings from Yu et al. and Ruiz et al. that burnout negatively correlates with academic performance [10,9]. Stress is closely related to academic burnout [10,19].

However, stress levels in the pre-clerkship and clerkship phases of medical school were not found to have a relationship with semester exams or Step 2, respectively. The fact that the pre-clerkship semester and Step 2 exams are the most initial and terminal exams in medical school, respectively, can potentially clarify this finding. The potentiation of stress may have not reached an effective threshold at the beginning of medical school to affect performance during the pre-clerkship semester exams. Our study shows that stress is 0.29 points higher in the clerkship phase compared to the pre-clerkship phase, although not statistically significant (p = 0.105). Other studies also show higher stress in later medical school years [4]. Similarly, grit near the end of standardized medical school examinations, which is well established to positively correlate with academic performance, may have been able to overcome the negative effects of stress on academic performance during Step 2 [20].

Furthermore, we found that relationships between wellness activity time and academic performance did not reach statistical significance. However, the earlier established relationships in our study between wellness activity time and stress levels may imply an indirect relationship to academic performance. It is a factor that cannot be ignored when optimizing a student’s academic performance.

Study limitations

Data collection was from a single medical school with a student demographic that may not reflect other medical schools. Although there is nothing that makes Kirk Kerkorian School of Medicine specifically unique that would notably influence the results, this fact could still limit the external validity of the results of this study. Second, the survey responses are subject to self-reporting and recall bias. Another limitation of this study was its inability to parse out the chronology of the factors. It is not known if it is stress that affects academic performance or if in fact, academic performance is affecting stress. Similarly, it is unclear if wellness activity time affects stress or vice versa. Additionally, this study did not explore other factors that could affect stress in medical students, such as their socioeconomic status, relationships with their peers, or the mentorship they may have.

## Conclusions

Medical students at the Kirk Kerkorian School of Medicine had a lower perceived stress scale score when they spent more time per week doing wellness activities in the pre-clerkship and clerkship phases of medical school. Students with lower stress levels during the pre-clerkship phase of medical school performed better on the USMLE Step 1 exam when compared to their peers with higher stress levels. Additionally, students with lower stress levels in the clerkship phase of medical school scored higher on their internal medicine, obstetrics/gynecology, and surgery clinical subject exams. Stress levels in the clerkship phase negatively correlated with the family medicine and psychiatry clinical subject exams. Similar results were found with the pre-clerkship semester 2 and 3 exams and Step 2, but they did not reach statistical significance. Although wellness activity time did not have a direct relationship with academic performance, its relationship with stress levels can possibly allude to an indirect effect on academic performance. This, along with fending off burnout and stress, are reasons why medical student wellness should be a priority for medical schools, faculty, and their students.

## References

[1] American Psychological Association (2024). Stress in America 2023: a nation recovering from collective trauma. https://www.apa.org/news/press/releases/stress/2023/collective-trauma-recovery.

[2] Dyrbye LN, Thomas MR, Massie FS (2008). Burnout and suicidal ideation among U.S. medical students. Ann Intern Med.

[3] Dyrbye LN, Thomas MR, Huntington JL, Lawson KL, Novotny PJ, Sloan JA, Shanafelt TD (2006). Personal life events and medical student burnout: a multicenter study. Acad Med.

[4] Ishak W, Nikravesh R, Lederer S, Perry R, Ogunyemi D, Bernstein C (2013). Burnout in medical students: a systematic review. Clin Teach.

[5] Stewart SM, Lam TH, Betson CL, Wong CM, Wong AM (1999). A prospective analysis of stress and academic performance in the first two years of medical school. Med Educ.

[6] Dyrbye LN, Thomas MR, Harper W (2009). The learning environment and medical student burnout: a multicentre study. Med Educ.

[7] Guthrie EA, Black D, Shaw CM, Hamilton J, Creed FH, Tomenson B (1995). Embarking upon a medical career: psychological morbidity in first year medical students. Med Educ.

[8] MacLeod RD, Parkin C, Pullon S, Robertson G (2003). Early clinical exposure to people who are dying: learning to care at the end of life. Med Educ.

[9] Yu JH, Chae SJ, Chang KH (2016). The relationship among self-efficacy, perfectionism and academic burnout in medical school students. Korean J Med Educ.

[10] Ruiz J, Kaminnik P, Kibble J, Kauffman C (2024). Relationships between medical student wellness, self-efficacy, and academic performance during the "post-COVID" period. Adv Physiol Educ.

[11] Drolet BC, Rodgers S (2010). A comprehensive medical student wellness program - design and implementation at Vanderbilt School of Medicine. Acad Med.

[12] House JD (2002). The independent effects of student characteristics and instructional activities on achievement: an application of the input-environment-outcome assessment model. Int J Instr Media.

[13] Credé M, Kuncel NR (2008). Study habits, skills, and attitudes: the third pillar supporting collegiate academic performance. Perspect Psychol Sci.

[14] Al Shawwa L, Abulaban AA, Abulaban AA (2015). Factors potentially influencing academic performance among medical students. Adv Med Educ Pract.

[15] Toews JA, Lockyer JM, Dobson DJ (1997). Analysis of stress levels among medical students, residents, and graduate students at four Canadian schools of medicine. Acad Med.

[16] Cohen S, Kamarck T, Mermelstein R (1983). A global measure of perceived stress. J Health Soc Behav.

[17] Hill MR, Goicochea S, Merlo LJ (2018). In their own words: stressors facing medical students in the millennial generation. Med Educ Online.

[18] Leuchter RK, Stuber ML, McDonald AL, Croymans DM (2022). Relationship between exercise intensity and stress levels among U.S. medical students. Med Educ Online.

[19] Chun KH, Park YS, Lee YH, Kim SY (2014). Academic burnout and selection-optimization-compensation strategy in medical students (Article in Korean). Korean J Med Educ.

[20] Luthans KW, Luthans BC, Chaffin TD (2019). Refining grit in academic performance: the mediational role of psychological capital. J Manage Educ.

